# Immune cell and TCR/BCR repertoire profiling in systemic lupus erythematosus patients by single-cell sequencing

**DOI:** 10.18632/aging.203695

**Published:** 2021-11-12

**Authors:** Fengping Zheng, Huixuan Xu, Cantong Zhang, Xiaoping Hong, Dongzhou Liu, Donge Tang, Zuying Xiong, Yong Dai

**Affiliations:** 1 Department of Nephrology, Peking University Shenzhen Hospital, Shenzhen Peking University-The Hong Kong University of Science and Technology Medical Center, Shenzhen 518020, Guangdong, China; 2 Clinical Medical Research Center, Guangdong Provincial Engineering Research Center of Autoimmune Disease Precision Medicine, Shenzhen Engineering Research Center of Autoimmune Disease, The Second Clinical Medical College, Shenzhen People's Hospital, Jinan University, Shenzhen 518020, Guangdong, China; 3 Department of Rheumatology and Immunology, The Second Clinical Medical College, Shenzhen People's Hospital, Jinan University, Shenzhen 518020, Guangdong, China

**Keywords:** SLE, single-cell sequencing, immune cells, TCR, BCR

## Abstract

The immune cells and the repertoire of T cells and B cells play an important role in the pathogenesis of systemic lupus erythematosus (SLE). Exploring their expression and distribution in SLE can help us better understand this lethal autoimmune disease. In this study, we used a single-cell 5’ RNA sequence and single-cell T cell receptor (TCR)/B cell receptor (BCR) to study the immune cells and the repertoire from ten SLE patients and the paired normal controls (NC). The results showed that 9732 cells correspondence to 12 cluster immune cell types were identified in NC, whereas 11042 cells correspondence to 16 cluster immune cell types were identified in SLE. The results demonstrated that neutrophil, macrophage, and dendritic cells were accumulated in SLE by annotating the immune cell types. Besides, the bioinformatics analysis of differentially expressed genes (DEGs) in these cell types indicates their role in inflammation response. In addition, patients with SLE showed increased TCR and BCR clonotypes compared with the healthy controls. Furthermore, patients with SLE showed biased usage of TCR and BCR V(D)J genes. Taken together, we characterized the transcriptome and TCR/BCR immune repertoire profiles of SLE patients, which may provide a new avenue for the diagnosis and treatment of SLE.

## INTRODUCTION

SLE is a lethal autoimmune disease caused by unknown reasons [1, 2]. Abnormalities in the regulation of cell-mediated immunity have been implicated in the pathophysiology of SLE [3]. For example, Bratha S. Devi et al. [3] found that the lack of T lymphocytes in the peripheral may contribute to the pathogenesis of SLE. Besides, B cells are an essential player in the pathogenic mechanism of SLE. Hanan Hassan Omar et al. [4] reported that CD5+ B cells were significantly decreased in SLE patients compared to healthy control. Furthermore, Hanan Hassan Omar et al. [4] also found that CD5+ B cells were significantly reduced in the active SLE patients compared to inactive ones, indicated that the numbers of CD5+ B cells in the peripheral might be related to the SLE disease activity. Interleukin (IL)-2 was markedly reduced the disease activity in SLE patients. The effect of IL-2 may refer to it selectively modulated the abundance of regulatory T (Treg) cell, follicular helper T (T_FH_) cells, and IL-17-producing helper T (T_H_17) cells, but not T_H_1 or T_H_2 cells [5]. The homeostasis of the lymphocyte’s subset is vital for an immune response [6, 7]. Therefore, having an overview of different cell types between SLE and healthy control can help us better understand its pathogenesis.

The diversity of TCR and BCR is also involved in determining the autoimmune response [8, 9]. Generally, the variety of TCR and BCR is achieved by the rearrangement of the variable (V), diversity (D), and Joining (J) genes at complementary-determining region 3 (CDR3) recombination junctions. Identifying disease-associated TCR and BCR can potentially serve as biomarkers and provide novel insights for disease status and therapeutic targets in SLE [10].

Single-cell sequencing has emerged as a robust new set of technologies for delineating complex populations [11–13]. It can elucidate the cell type composition of a sample. For example, Dominic Grun et al. [14, 15] discovered a rare intestinal cell type using single-cell mRNA sequencing. The heterogeneity of SLE can be revealed by a single-cell RNA sequence [14, 15]. Here, we used single-cell 5’ RNA sequencing and single-cell repertoire sequencing to reveal the cell types in peripheral blood of SLE patients, elucidate their biological process in the pathogenesis of SLE, and discover the disease-associated TCR/BCR.

## MATERIALS AND METHODS

### Ethics statement

The experimental study was approved by the ethics committee of Shenzhen people’s hospital (LL-KT-2018358). The study adheres to the Helsinki Declaration guidelines on ethical principles for medical research involving human subjects.

### Patient involvement

Ten peripheral blood samples of SLE patients and Ten NC samples were obtained from Shenzhen People's Hospital. Inclusion criteria: Patients diagnosed with SLE and SLE disease activity index (SLEDAI) scores are equal to or greater than 5 (Table 1). Exclusion criteria: patient and other immune diseases or treated with high dose (>1mg/kg/d) glucocorticoid and other immunosuppressants. All participants have signed the informed consent form.

**Table 1:** Clinical data of participants.

**Group**	**Age**	**Proteinuria(g/24h)**	**C3(g/l)**	**C4(g/l)**	**ESR**	**SLEDAI**
SLE	45±12.3	1.3±1.35	0.65±0.37	0.15±0.03	58.9±15.3	5.8±0.5
NC	42±11.8	0.06±0.02	1.83±0.62	0.4±0.1	9.8±2.7	0

C3, complement 3; C4, complement 4; ESR, erythrocyte sedimentation rate; h, hours; NC, normal control; SLE, systemic lupus erythematosus, SLEDAI, systemic lupus erythematosus disease activity index.

### Peripheral blood monocytes (PBMCs) isolation

The PBMCs were isolated by using a density gradient centrifugation method. Briefly, a blood sample from an ethylenediaminetetraacetic acid-anticoagulated tube was added to Ficoll paque plus density solution (17-1440- 03,GE Healthcare) and then centrifuged according to the manufacturer’s protocol.

### Single-cell 5’ RNA sequencing

After PBMCs pooling, the suspension was loaded onto a Chromium platform (10x Genomics). The single-cell 5’ RNA sequencing as described in the previous publication [16]. In brief, we profiled thousands of genes at the single cell level by barcoding mRNA at the 5’ end, for unbiased characterization of cell types and cell states.

### TCR/BCR profiling

At the same time, as we previously described, samples were processed per Chromium single cell V(D)J reagent kits protocol by 10x Genomics [17, 18]. We simultaneously profiled immune repertoire (BCR/TCR) and gene expression from the same cell to enable the correlation of clonotype with the corresponding cell subtype.

### Data analysis

All raw data were processed through the CellRanger pipeline (10x Genomics), which allowed demultiplexing, alignment, filtering, barcode counting, UMI counting, and generating cell x barcode matrices. Unsupervised clustering and visualization were performed with R and t-distributed Stochastic Neighbor Embedding (tSNE), respectively. Marker genes (Supplementary Table 1) were defined with fold change adjusted p <0.05. Cell clusters were annotated using canonical markers by cellmarker [19] (http://bio-bigdata.hrbmu.edu.cn/CellMarker/). The Gorilla tool for discovering enriched GO terms in the ranked gene list was used to determine transcriptional signatures. For TCR/BCR clonotype analysis, we used the CellRanger pipeline. As per the definition by 10x genomics: “clonotypes are defined as a set of cells with the same CDR3 sequence in their V(D)J variable regions”.

### Availability of data and materials

All data can be available in this manuscript, supplementary information files, and the corresponding author upon request.

## RESULTS

### Abnormal subsets of neutrophil, macrophages, and dendritic cells were accumulated in the peripheral blood of SLE patients

**Figure 1:**
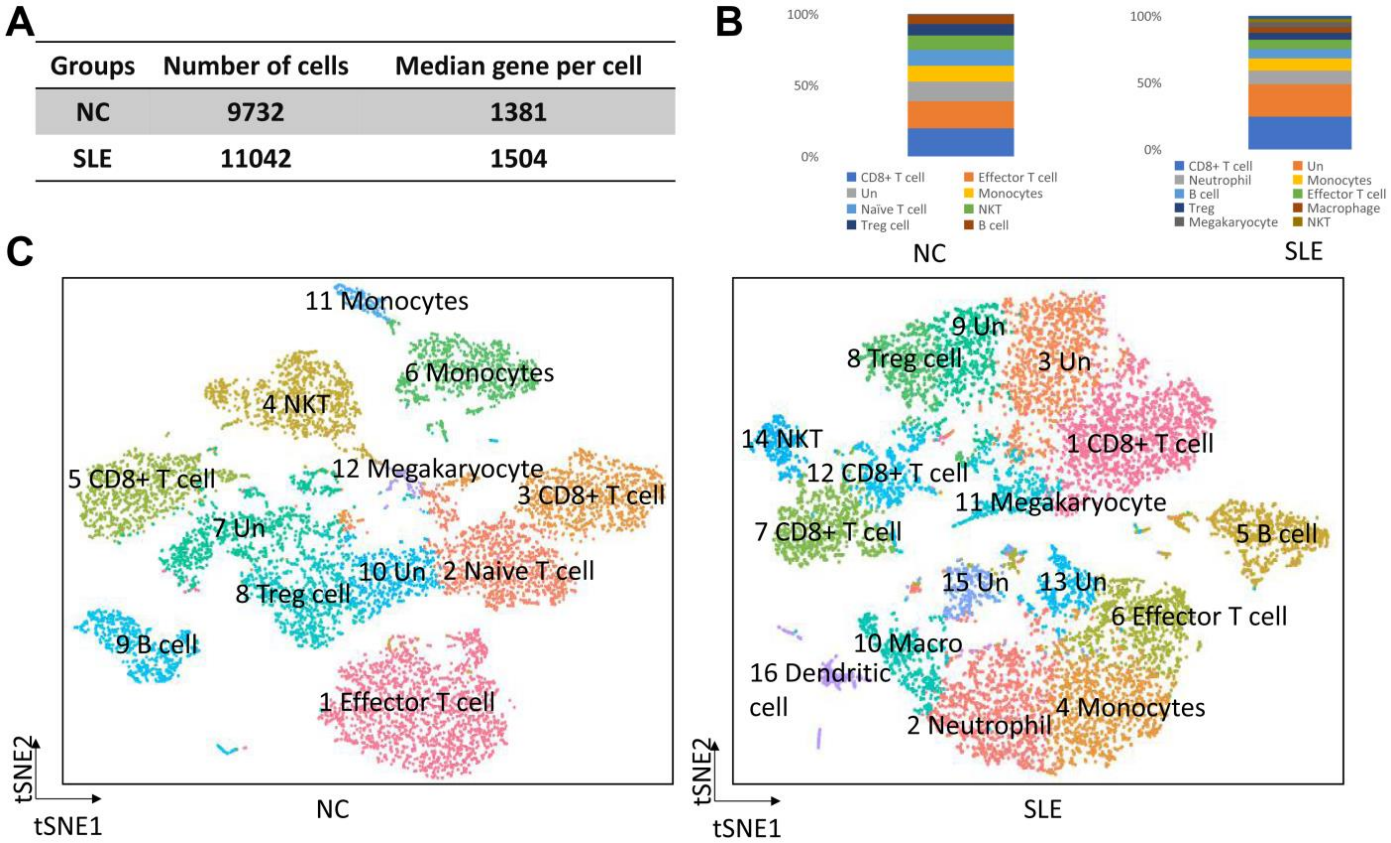
**The overview of clusters in the SLE and NC.** The accumulation of neutrophil, macrophage, and dendritic cells in SLE. (**A**) The cell number and median gene per cell were generated in NC and SLE. (**B**) The bar chart showed the immune cell types in NC and SLE. (**C**) The t-distributed stochastic neighbor embedding (t-SNE) analysis of cell clusters between NC and SLE. Un: undefined cell types, macro: macrophage cells.

We generated different cell numbers in SLE patients (11042) and NC (9732) (Figure 1A). In SLE, the mean reads per cell are 38294, and the median gene per cell is 1504. However, the mean reads per cell are 42599, and the median gene per cell is 1381 (Supplementary Figure 1) in NC. We further identified 12 cell clusters among 9732 cells in the NC group and 16 cell clusters among 11042 cells in the SLE group. Next, we defined these cell clusters by gene marker. Compared to the NC group, the neutrophil, macrophage, and dendritic cells accumulated in the SLE group (Figure 1B, 1C), indicating that these cells clusters might play an important role in the inflammation response. In addition, the trajectory analysis results showed five states in cell clusters of SLE groups. In contrast, only three states were showed in NC groups. The results suggested a different lineage hierarchy among the major immune cell types of PBMCs between these two groups (Supplementary Figure 2). Therefore, the roles of each cell type were different in NC and SLE.

### The role of neutrophil cells in the pathogenesis of SLE

**Figure 2:**
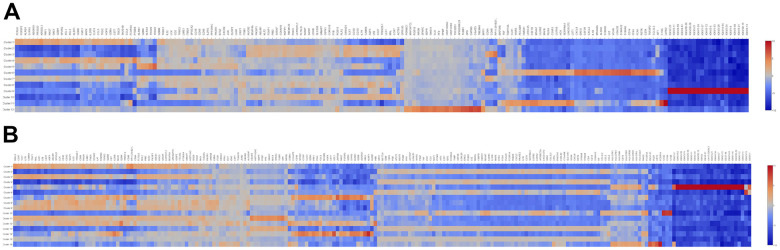
**The heatmap of DEGs in different cell clusters between the SLE and NC groups.** (**A**) The heatmap of DEGs in 12 cell clusters of the NC group. (**B**) The heatmap of DEGs in 16 cell clusters of the SLE group.

**Figure 3:**
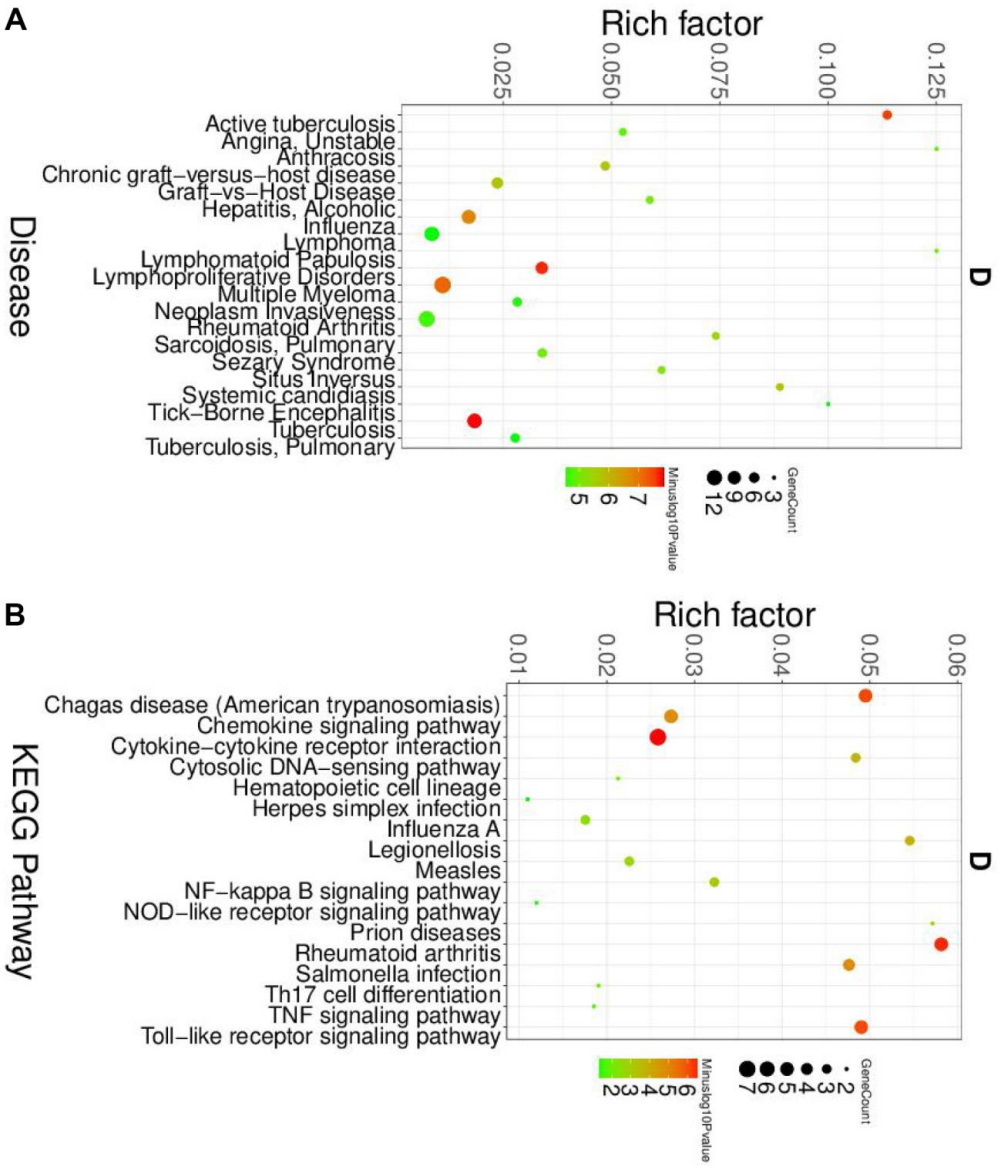
**The disease and KEGG enriched of DEGs in neutrophils.** (**A**) The top disease enriched of DEGs in neutrophils. (**B**) The KEGG pathway analyses of DEGs in neutrophils.

To further elucidated the role of neutrophil, macrophage, and dendritic cells in the pathogenesis of SLE, we performed the gene ontology (GO) and Kyoto Encyclopedia of Genes and Genomes (KEGG) pathway analysis. First, the differential expression genes (DEGs) in each cluster were identified. The heatmap of the DEGs showed in Figure 2. The disease enriched results showed that the DEGs in neutrophils play a role in autoimmune diseases, such as rheumatoid arthritis and lymphoproliferative disorders (Figure 3A). The GO analysis results further confirmed that the biological process of DEGs was enriched by the immune system process (Supplementary Figure 3). The KEGG pathway results showed that the DEGs in neutrophils might, through the NF-κB pathway, TNF signaling pathway participated in the pathogenesis of SLE (Figure 3B). These results suggested that neutrophils have an important role in the pathogenesis of SLE.

### The role of macrophage and dendritic cells in the pathogenesis of SLE

**Figure 4:**
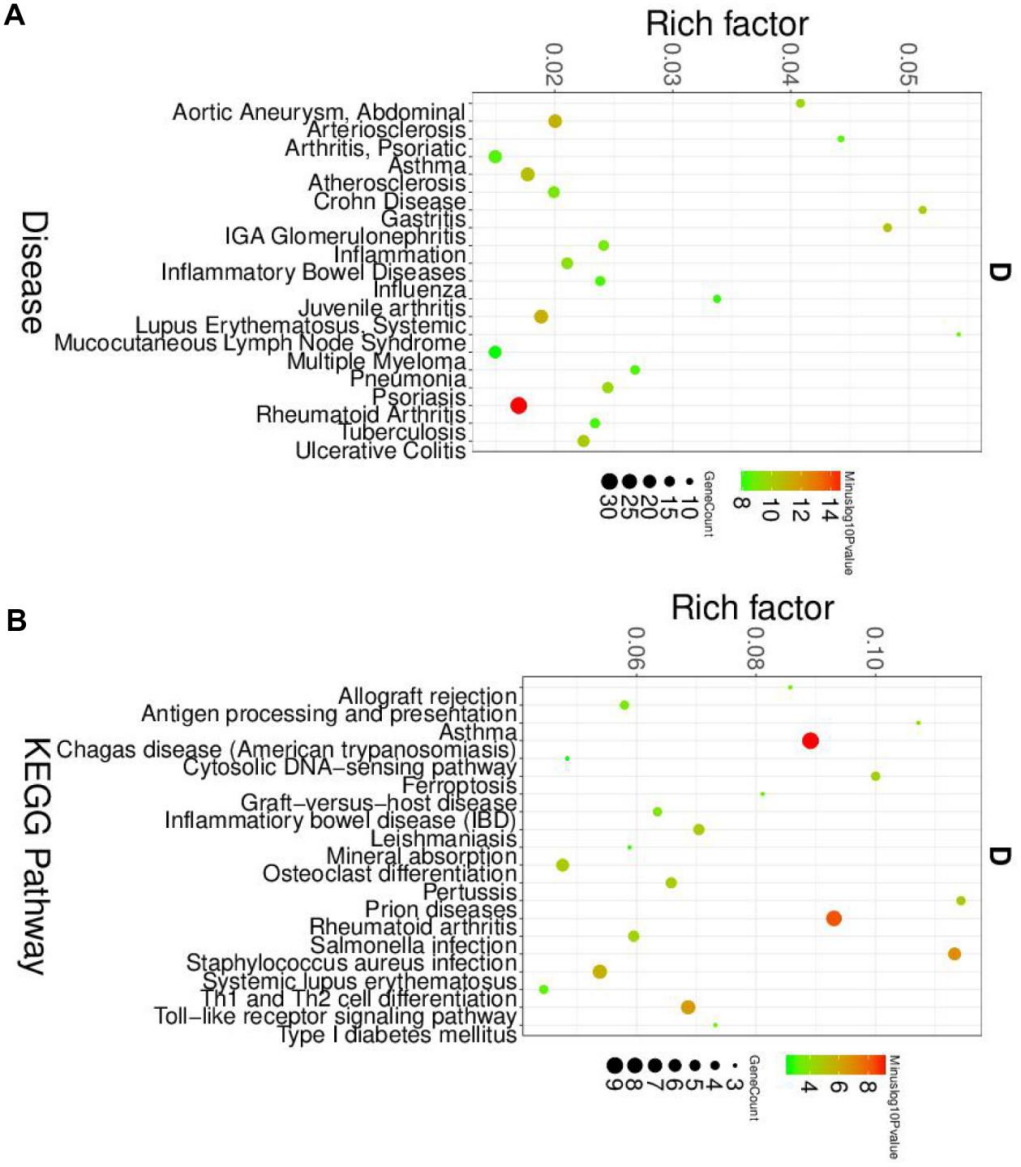
**The disease and KEGG enriched of DEGs in macrophage cells.** (**A**) The top disease enriched of DEGs in macrophage cells. (**B**) The KEGG pathway analyses of DEGs in macrophage cells.

Next, we analyzed the roles and macrophage and dendritic cells in the pathogenesis of SLE. Similar to the neutrophil, the GO and KEGG analyses were performed. The disease enriched analysis showed that the DEGs in macrophages were directly involved in the SLE (Figure 4A), and the results were further confirmed by the GO analysis results (Supplementary Figure 4). The KEGG results showed that the DEGs in macrophages would affect the Th1 and Th2 cell differentiation (Figure 4B). The differentiation status of Th1 and Th2 cells was correlated with the SLE [20]. Therefore, the DEGs in macrophage plays a vital role in the pathogenesis of SLE.

**Figure 5:**
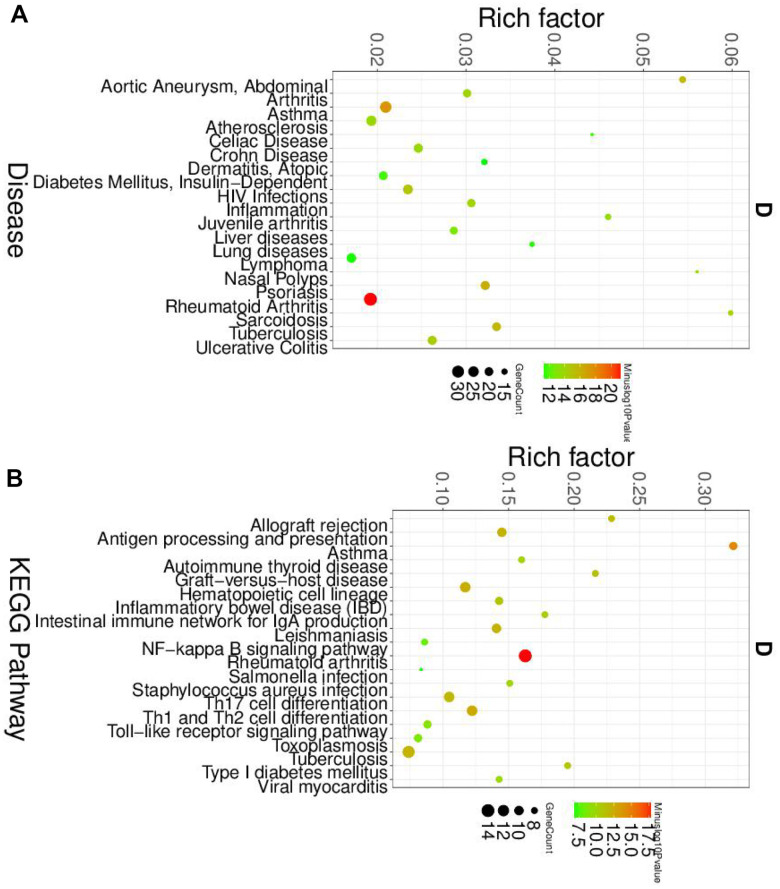
**The disease and KEGG enriched of DEGs in dendritic cells.** (**A**) The top disease enriched of DEGs in dendritic cells. (**B**) The KEGG pathway analyses of DEGs in dendritic cells.

Furthermore, the DEGs of dendritic cells were also enriched by the autoimmune disease (Figure 5A) and immune response (Supplementary Figure 5). The KEGG pathway results showed that dendritic cells' DEGs were involved in the NF-κB pathway and Th1 and Th2 cell differentiation (Figure 5B).

Overall, our finding demonstrated that different immune cell types participated in the immune response in SLE through different pathways, which helped us better understand the pathogenesis of SLE.

### The TCR clonotypes were increased in the SLE

**Figure 6:**
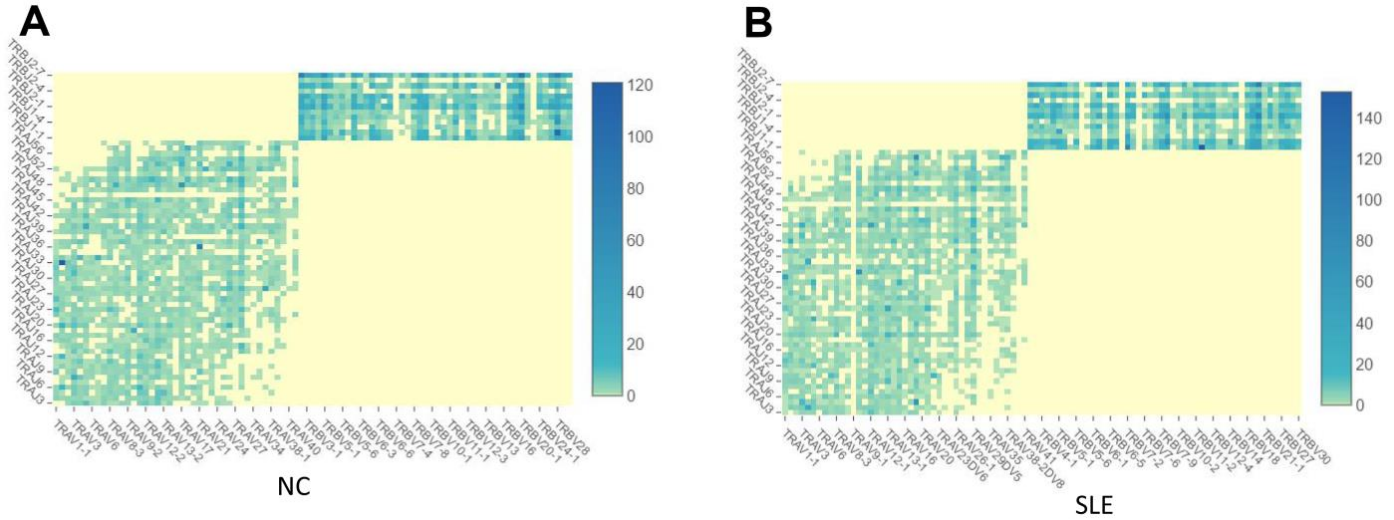
**The TCR clonotypes was increased in SLE than in NC.** (**A**) The heatmap of TCR VDJ in the NC group. (**B**) The heatmap of TCR VDJ in the SLE group.

**Figure 7:**
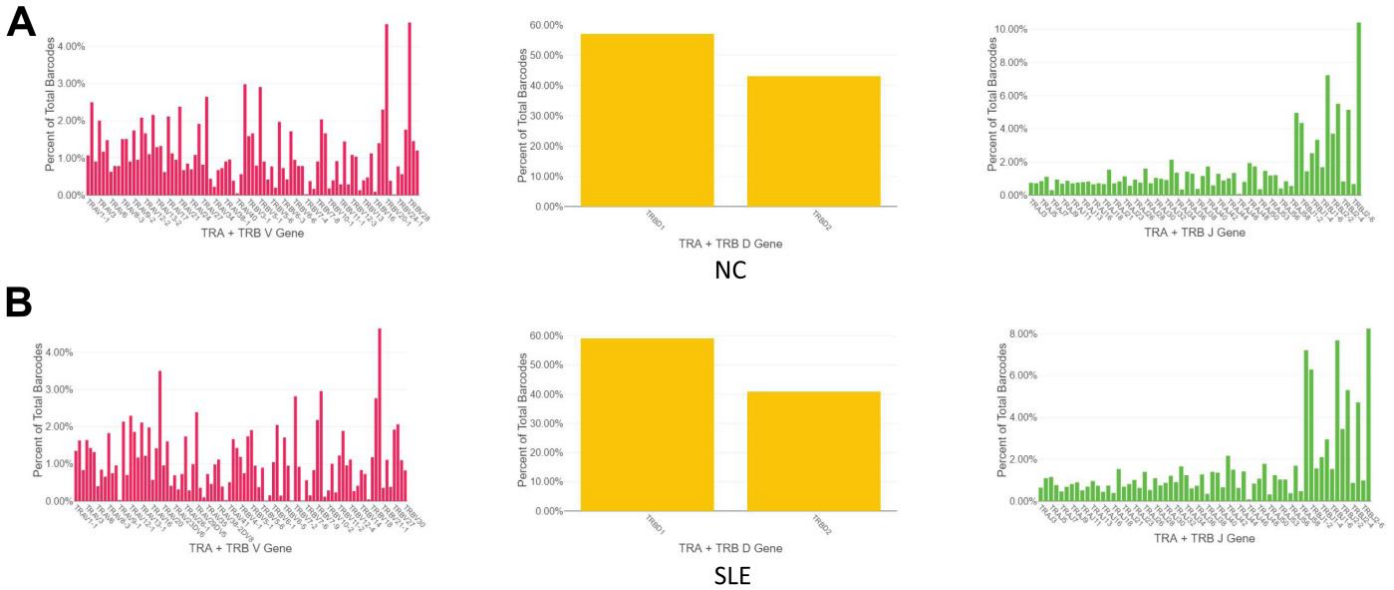
**The biased usage of the VDJ gene of TCR between SLE and NC group.** (**A**) The usage of the VDJ gene of TCR α and β chain in NC group. (**B**) The usage of the VDJ gene of TCR α and β chain in the SLE group.

The immune repertoire can be used for either diagnostic marker or therapeutic targets. Using the Cell Ranger pipeline, we identified 5167 clonotypes from 5847 barcodes in SLE patients, 4259 clonotypes from 4654 barcodes in NC, suggesting that the TCR clonotypes was increased in the SLE compared to NC (Figure 6). In addition, the usage of the VDJ gene in both TCR α and β chains was biased between these two groups (Figure 7). For example, the most used V gene in TCRα was TRAV40 in NC, whereas it was TRAV16 in SLE. The most usage TCR clonotype was shown in Supplementary Figure 6A (CALSGDSNYQLIW_CASSPRPGNTEAFF).

### The BCR clonotypes were increased in the SLE

**Figure 8:**
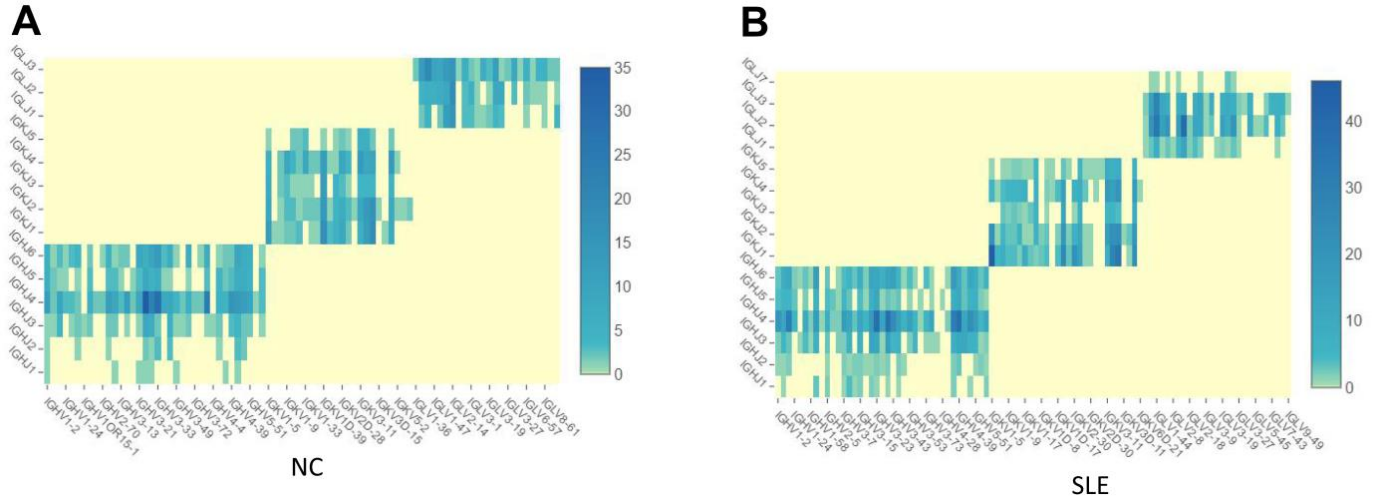
**The BCR clonotypes was increased in SLE than in NC.** (**A**) The heatmap of BCR in the NC group. (**B**) The heatmap of BCR in the SLE group.

**Figure 9:**
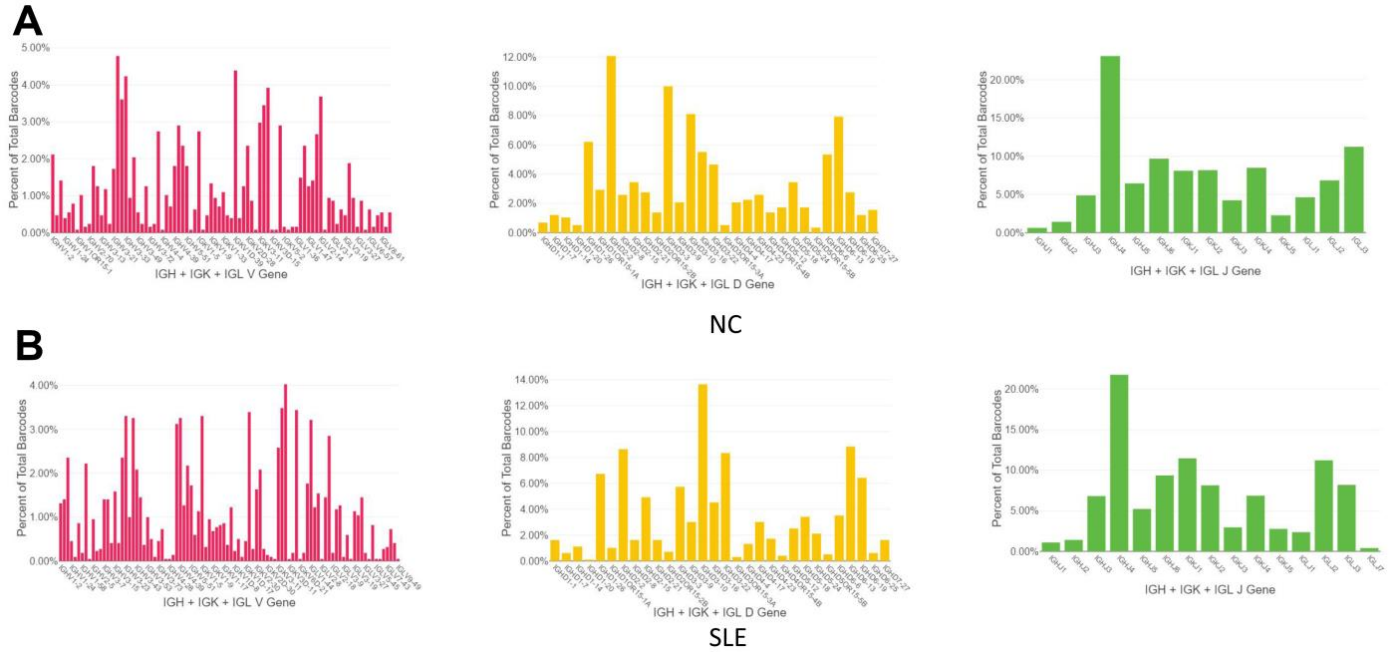
**The biased usage of the VDJ gene of BCR between NC and SLE group.** (**A**) The usage of VDJ gene of BCR IGH, IGK, and IGL chain in the NC group. (**B**) The usage of the VDJ gene of BCR IGH, IGK, and IGL chain in the SLE group.

1064 clonotypes from 1087 barcodes were identified in SLE, whereas 639 clonotypes from 653 barcodes were identified in NC. The results showed that the BCR clonotypes was also increased in the SLE compared to NC (Figure 8 and Supplementary Figure 7). In addition, the usage of the VDJ gene in both BCR IGH, IGK, and IGL chains was biased between these two groups (Figure 9). For example, the most use of V gene in BCR IGH was IGHV3-21 in NC, whereas it was IGHV3-23 in SLE. The most usage BCR clonotype was shown in Supplementary Figure 6B (CAAWDASLNGGWVF_CARGVAALW). The different usage of TCR and BCR may provide us an avenue to develop a specific treatment for SLE patients.

## DISCUSSION

In this study, we revealed different immune cell types between the SLE and NC. The hemostasis subsets and populations of lymphocytes are important in the maintenance of immune systems. Abnormal cell subsets in peripheral blood may lead to immune-related diseases, such as SLE, rheumatoid arthritis, and IgA nephritis. In SLE, the abnormal T cell subsets may be involved in the pathogenesis of SLE [21].

In contrast to normal and inactive SLE patients, the percentage of typical T cell antigen-positive cells were reduced in all active SLE patients. In other words, the imbalance subsets of human T cells exist in patients with SLE [21]. Every T cell subsets have their specific role in SLE. The roles of CD4+ T cell, CD8+ T cell, Th1, Th2, Th17, and Treg cell in SLE have been well documented [20, 22–25].

This study first observed the accumulation of neutrophil, macrophage, and dendritic cells in SLE. And then, their roles in the pathogenesis of SLE were elucidated. The results showed that these three immune cell types participated in the pathogenesis through a different pathway. The correlation of neutrophil and SLC were investigated by a retrospective study. In this study, 154 SLE patients and 151 healthy controls were involved. The neutrophil to lymphocyte ratio (NLR) was positively correlated with C-reaction protein, SLE disease activity index (SLEDAI) scores, suggested an important role of neutrophil in SLE [26]. In addition, the formation of neutrophil extracellular traps indicated that the injury in the host [27].

The abnormal macrophage was observed in this study as well as other groups [28, 29]. In SLE patients, the macrophage might contribute to the acceleration of atherosclerosis [30], which is a severe cardiovascular disease. In addition to macrophages, dendritic cells are also important in regulating both immunity and tolerance. Generally, the population of dendritic cells in peripheral blood is very low. However, several cell types may differentiate into dendritic cells when responding to inflammatory status [31]. Patrick Blanco et al. [32] found that serum from SLE patients can initiate the differentiation from normal monocytes into dendritic cells. The capacity of SLE patient’s serum to generate dendritic differentiation related to disease activity. Accumulation shreds of evidence have highlighted dendritic cells as the culprit for SLE pathogenesis, mainly through type-I interferons production [33, 34]. Dendritic cells are hyperactivated in SLE patients who respond to rituximab, which requires a normalized plasmacytoid dendritic cell and regulatory B cell interaction [35]. Therefore, dendritic cells are crucial in both the innate and adaptive immune response. Either directly or via produced interferons, it has a pivotal role in autoimmunity. In addition, we also revealed the critical pathway of these cells involved, such NF-kB pathway and TNF signaling pathway. Their roles in the pathogenesis and the treatment of SLE have been reported [36–38].

The immune repertoire can be used for either diagnostic marker or therapeutic targets. The identified TCR/BCR can provide targets for chimeric antigen receptor (CAR)-T cell-based-adoptive immuno-therapies and neoantigen-specific TCR-T cell-based adoptive immuno-therapies [39]. Recently, Jin et al. [40] claimed that anti-CD19 CAR-T cell therapy worked effectively in treating murine SLE, suggested its potential in treating human patients. Besides, the disease-associated TCR/BCR can identify antibodies that contribute to functional immune response [41]. The TCR/BCR can be developed as diagnostic biomarkers or therapeutic targets in SLE [42]. These studies suggested that identifying disease-related TCR/BCR was critical for the diagnosis and treatment of SLE. This study revealed biased TCR/BCR in SLE patients and identified some frequent TCR/BCR clonotypes in SLE. We hope our study can provide some evidence to discover the SLE-related TCR/BCR. Further investigation is warranted.

## CONCLUSIONS

Our finding first elucidated the difference of immune cell subsets between SLE and NC. It then explored their transcription signature to have a better understanding of the pathogenesis of SLE. We also identified several TCR/BCR clonotypes, which can be developed as diagnostic biomarkers or therapeutic targets. However, the sample included in this study is SLEDAI greater than five, and the sample size is limited. Further study is necessary. Overall, we used single-cell RNA sequencing to reveal the transcriptome signature of immune cells and their immune repertoire in SLE in this study.

## Supplementary Material

Supplementary Figures

Supplementary Table 1
