# Supplementary material for: Immune cell and TCR/BCR repertoire profiling in systemic lupus erythematosus patients by single-cell sequencing
Source: Aging (Albany NY). 2021 Nov 12;13(21):24432–48. doi: 10.18632/aging.203695 (PMC8610142; doi:10.18632/aging.203695)
Supplement: Supplementary Figures [file aging-13-203695-s001.pdf]

SUPPLEMENTARY FIGURES

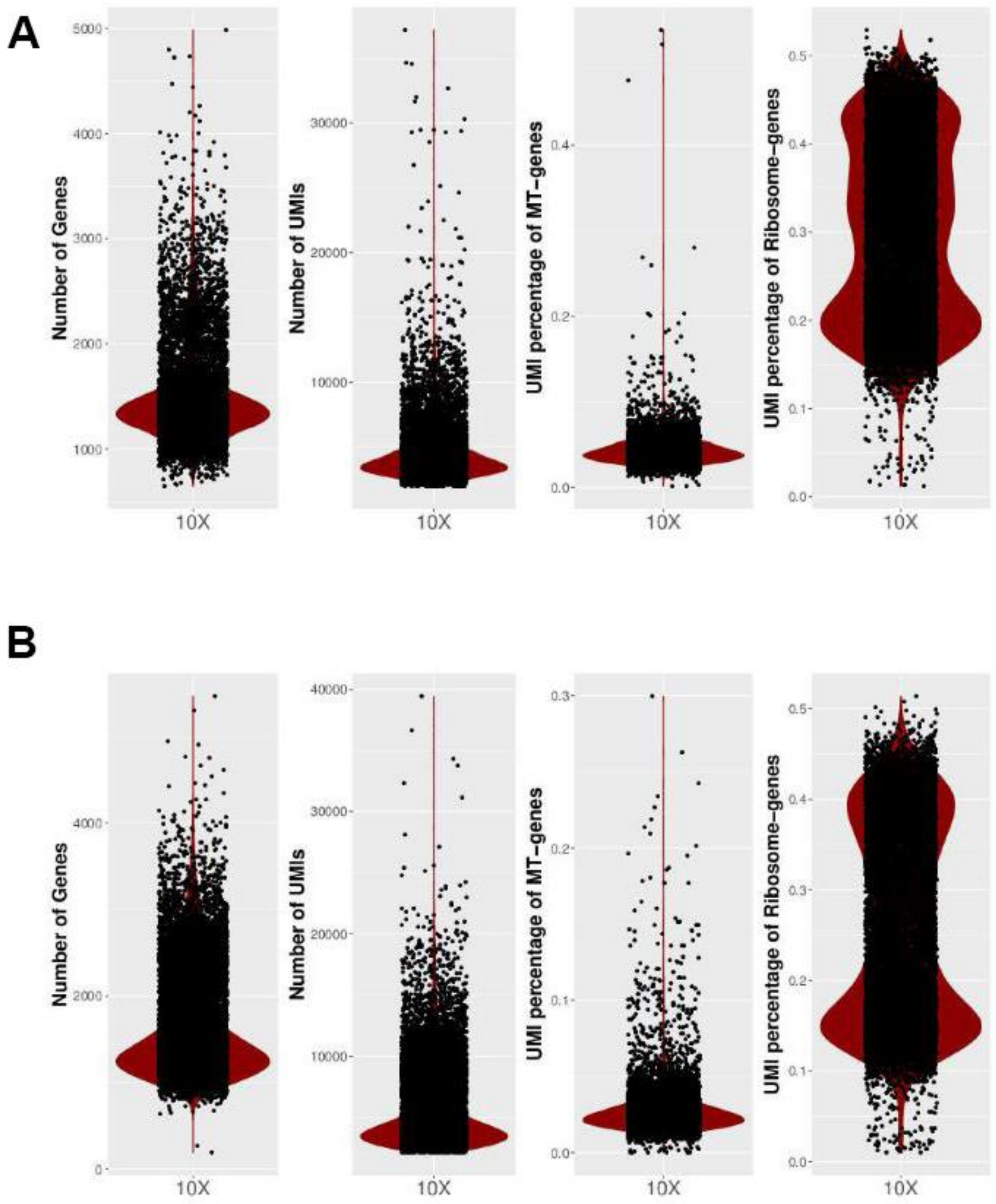

Supplementary Figure 1. The quality control of the single-cell sequencing. (**A**) NC group. (**B**) SLE group.

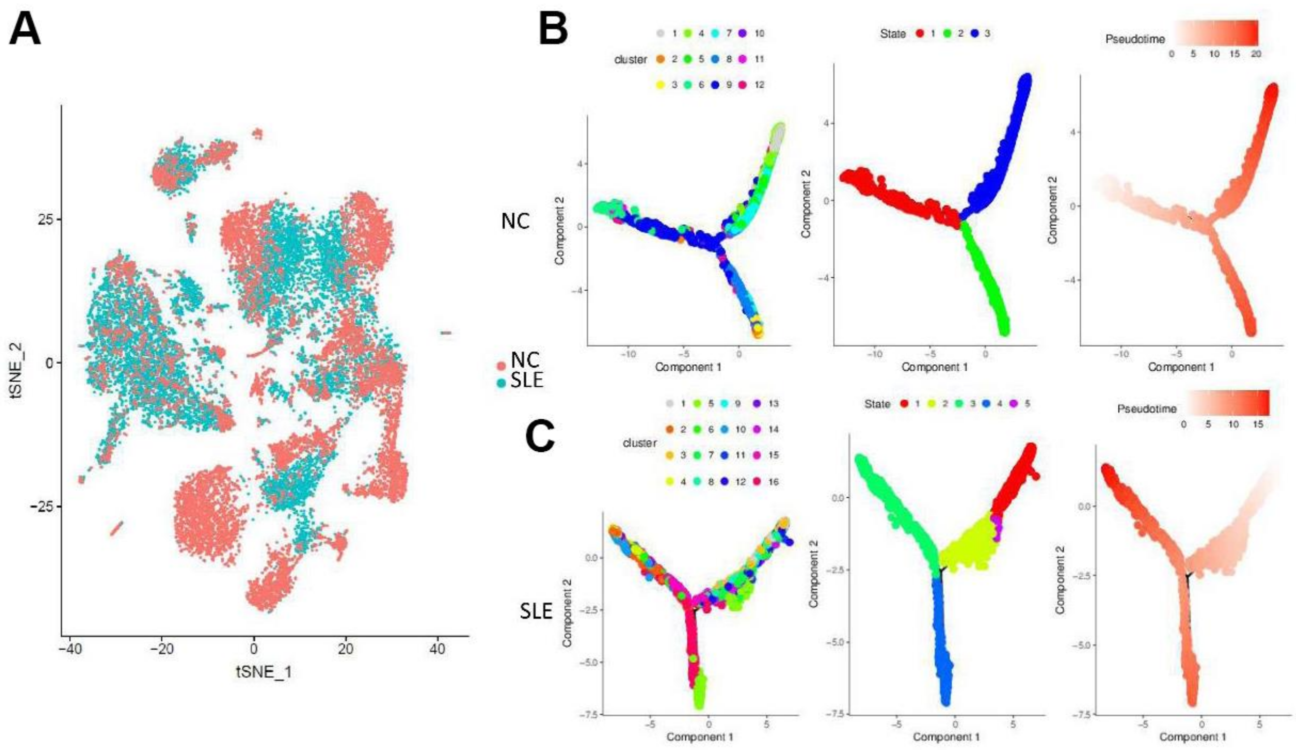

Supplementary Figure 2. (**A**) The integrative tSNE analysis of NC and SLE. (**B**) The trajectory analysis of NC group. **(C)** The trajectory analysis of SLE.

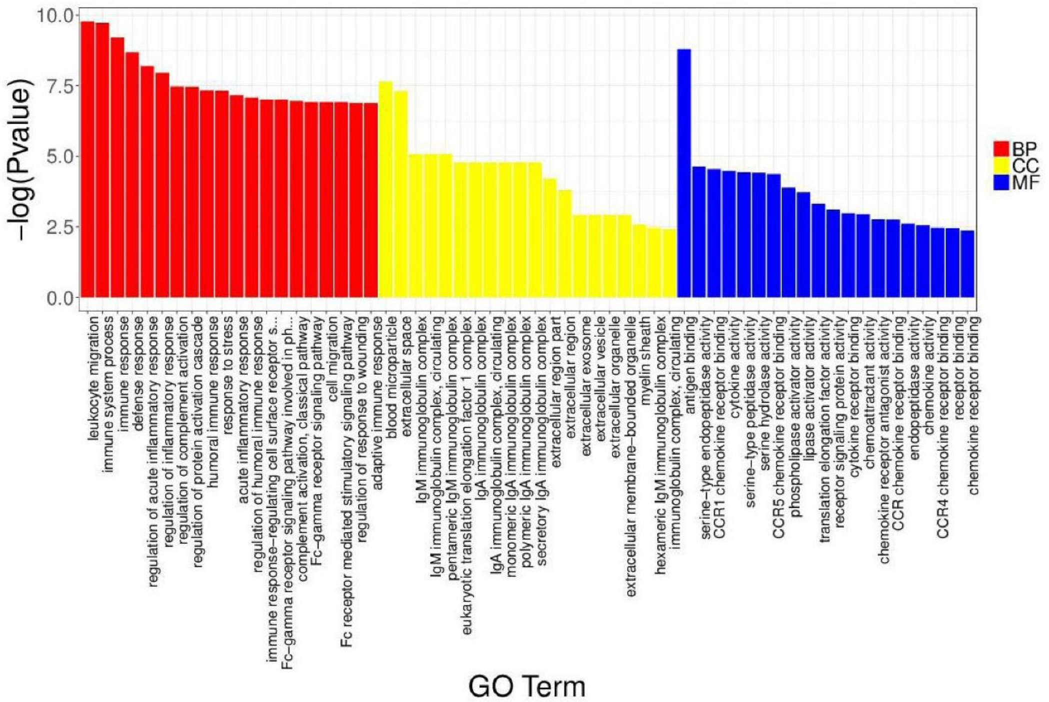

Supplementary Figure 3. GO analysis of DEGs in neutrophil accumulated in SLE group.

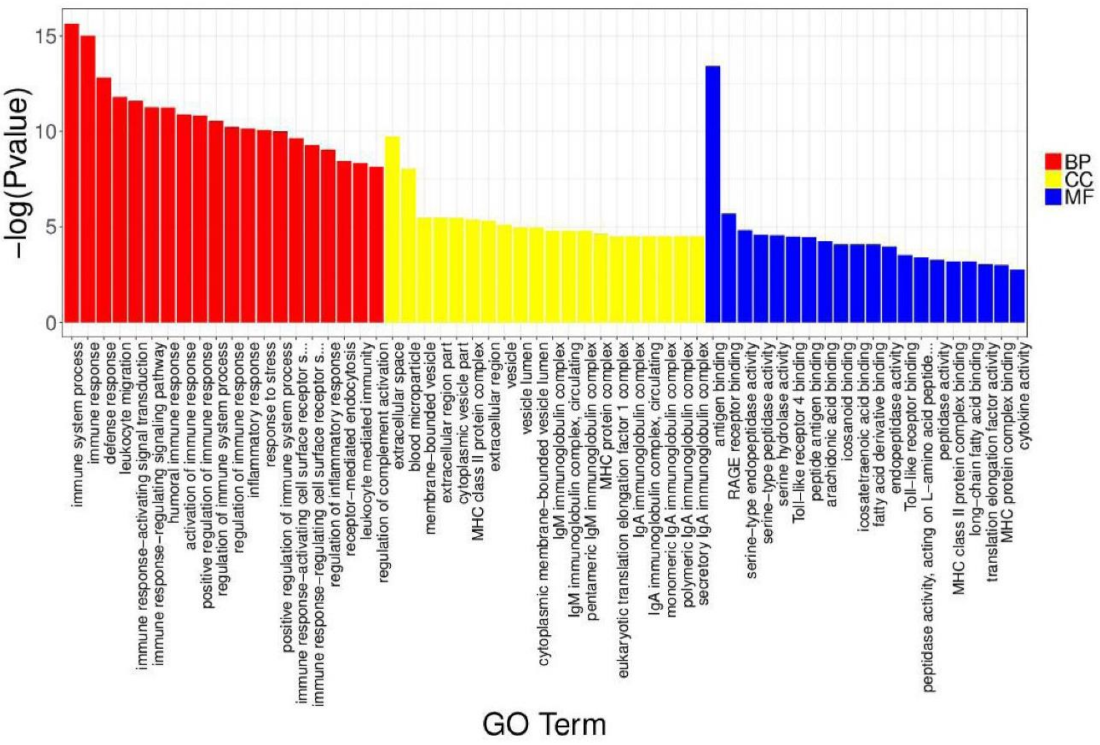

Supplementary Figure 4. GO analysis of DEGs in macrophage accumulated in SLE group.

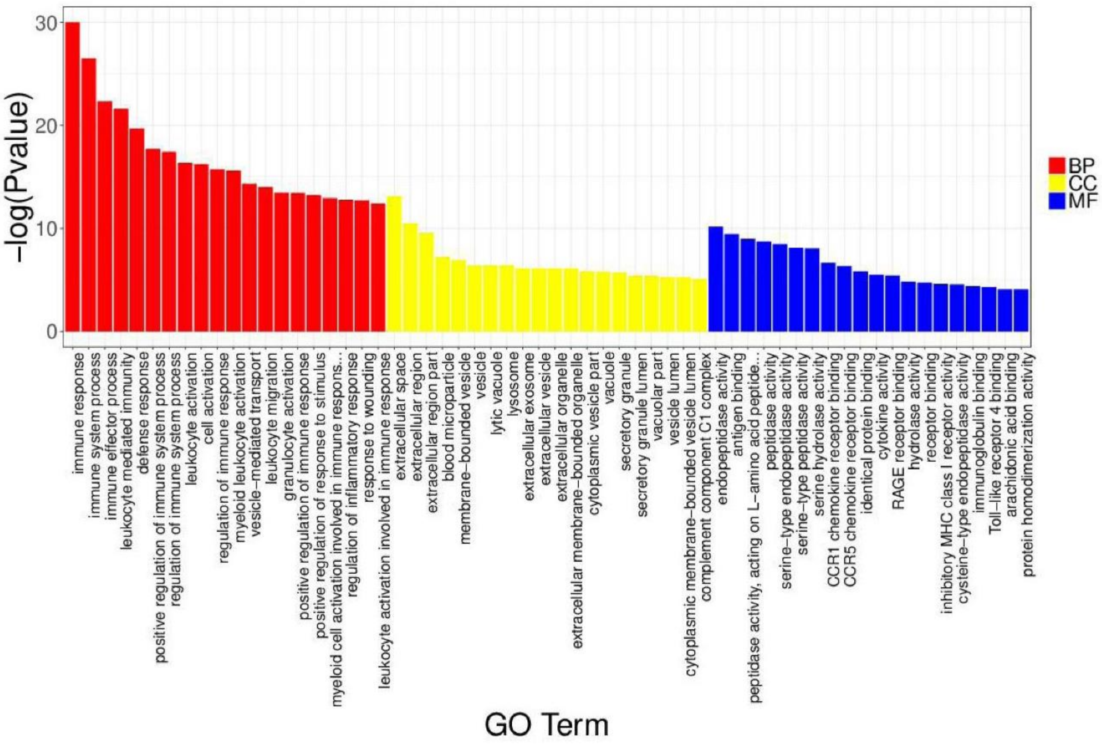

Supplementary Figure 5. The GO analysis of DEGs in dendritic cells in SLE group.

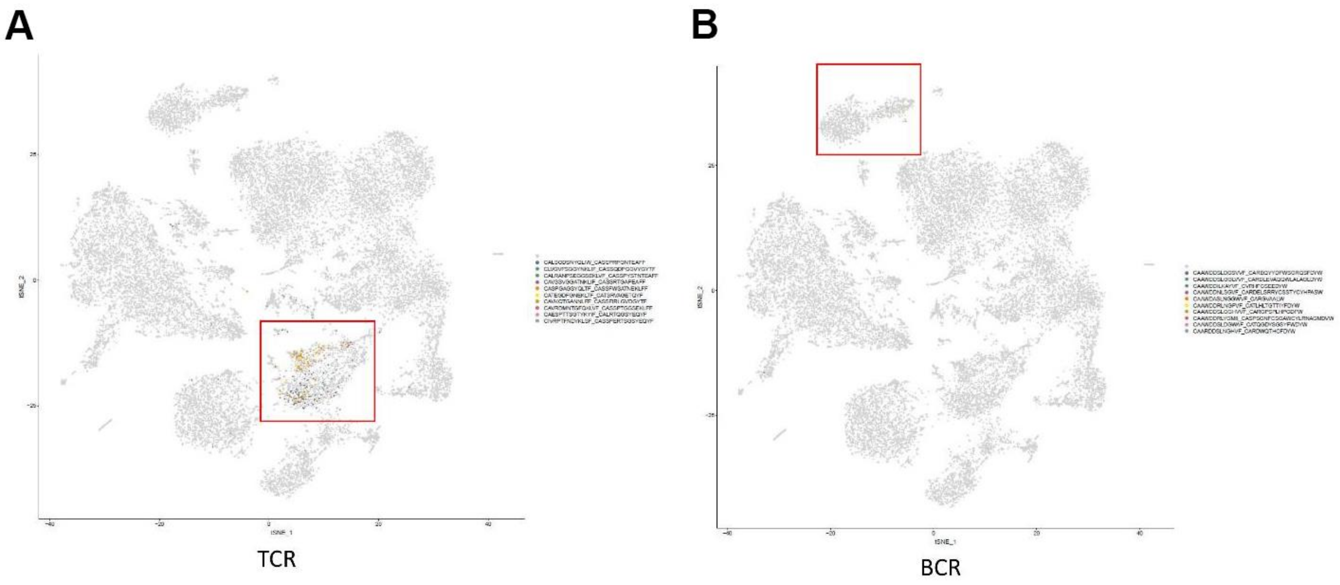

Supplementary Figure 6. The TCR (**A**) and BCR (**B**) matched in cell clusters.

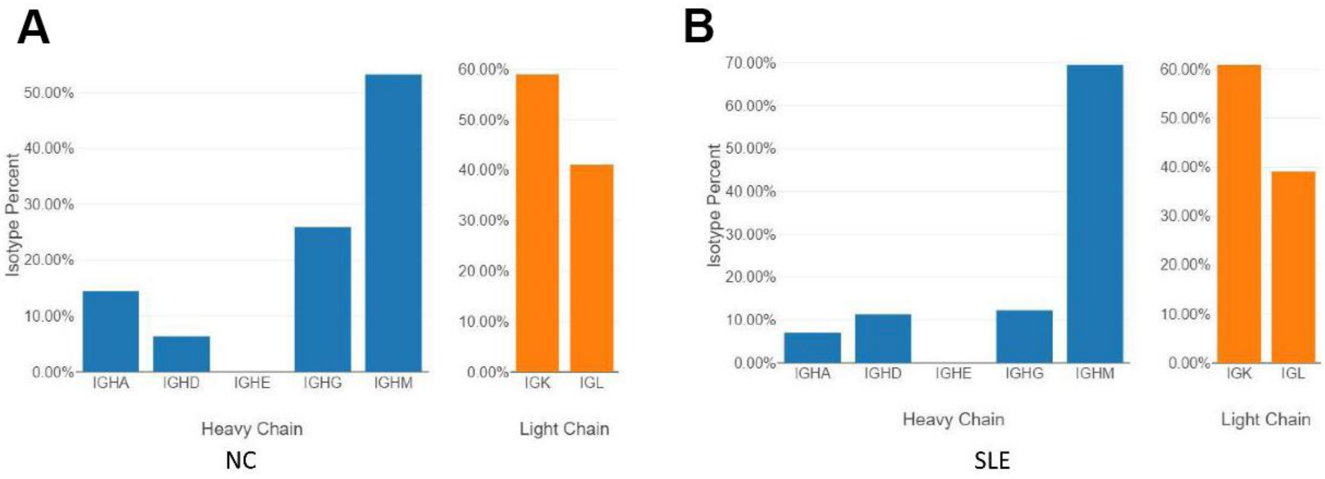

Supplementary Figure 7. The BCR isotypes in NC group (**A**) and SLE group (**B**).
